# Predicting the Responses of Soil Nitrite-Oxidizers to Multi-Factorial Global Change: A Trait-Based Approach

**DOI:** 10.3389/fmicb.2016.00628

**Published:** 2016-05-17

**Authors:** Xavier Le Roux, Nicholas J. Bouskill, Audrey Niboyet, Laure Barthes, Paul Dijkstra, Chris B. Field, Bruce A. Hungate, Catherine Lerondelle, Thomas Pommier, Jinyun Tang, Akihiko Terada, Maria Tourna, Franck Poly

**Affiliations:** ^1^UMR INRA 1418, UMR CNRS 5557, Microbial Ecology Centre, INRA, CNRS, Université Lyon 1, Université de LyonVilleurbanne, France; ^2^Earth Sciences Division, Lawrence Berkeley National Laboratory, BerkeleyCA, USA; ^3^UMR 8079, AgroParisTech, Ecology Systematics and Evolution Laboratory, CNRS, Université Paris-Sud 11Orsay, France; ^4^Ecosystem Science and Society Center, Department of Biological Sciences, Northern Arizona University, FlagstaffAZ, USA; ^5^Department of Global Ecology, Carnegie Institution, Stanford University, StanfordCA, USA; ^6^Department of Environmental Engineering, Technical University of DenmarkKongens Lyngby, Denmark

**Keywords:** bacterial functional traits, elevated CO_2_, nitrifiers, nitrogen fertilisation, trait-based modeling

## Abstract

Soil microbial diversity is huge and a few grams of soil contain more bacterial taxa than there are bird species on Earth. This high diversity often makes predicting the responses of soil bacteria to environmental change intractable and restricts our capacity to predict the responses of soil functions to global change. Here, using a long-term field experiment in a California grassland, we studied the main and interactive effects of three global change factors (increased atmospheric CO_2_ concentration, precipitation and nitrogen addition, and all their factorial combinations, based on global change scenarios for central California) on the potential activity, abundance and dominant taxa of soil nitrite-oxidizing bacteria (NOB). Using a trait-based model, we then tested whether categorizing NOB into a few functional groups unified by physiological traits enables understanding and predicting how soil NOB respond to global environmental change. Contrasted responses to global change treatments were observed between three main NOB functional types. In particular, putatively mixotrophic *Nitrobacter*, rare under most treatments, became dominant under the ‘High CO_2_+Nitrogen+Precipitation’ treatment. The mechanistic trait-based model, which simulated ecological niches of NOB types consistent with previous ecophysiological reports, helped predicting the observed effects of global change on NOB and elucidating the underlying biotic and abiotic controls. Our results are a starting point for representing the overwhelming diversity of soil bacteria by a few functional types that can be incorporated into models of terrestrial ecosystems and biogeochemical processes.

## Introduction

The structure and functioning of ecosystems are impacted by human activities (Steffen et al., 2011). Among key aspects of global change, altered precipitation, elevated atmospheric CO_2_ concentration, and rising nitrogen (N) deposition are occurring simultaneously and affect both resource availability and disturbance dynamics (Shaw et al., 2002; Zavaleta et al., 2003; Blankinship et al., 2011). Soil microorganisms have a strong influence on the responses of terrestrial ecosystems to global change and feedback to climate (Arrigo, 2004; Singh et al., 2010; Zhao et al., 2014). Many studies have reported the effects of single global change factors on the functioning of the soil microbiota, and fewer have reported the effects of multiple, interacting global change factors (Horz et al., 2003). Descriptive studies of environmental change effects on soil bacteria are, however, not sufficient to understand the mechanisms that determine their diversity and activity in soil. A major issue is that soils harbor a tremendous diversity of bacteria (Torsvik et al., 1998; Gans et al., 2005), and breaking down the soil bacterial diversity into functional units and predicting their responses to multifactorial global changes remains largely intractable. This impairs our ability to distil the diversity of soil microorganisms into discrete functional groups amenable to inclusion in ecosystem models representing global change effects (Wieder et al., 2013), which is a recognized need in microbial and ecosystem ecology (Prosser et al., 2007; Green et al., 2008).

In this context, trait-based approaches that reduce community complexity to key functional attributes (traits) can help microbial ecologists broaden the findings of empirical studies and develop a predictive framework for how biotic (e.g., microbial physiology) and abiotic (e.g., substrate concentration) variables interact to determine microbial fitness and community structure along environmental gradients (Shipley et al., 2006; Ackerly and Cornwell, 2007; Lavorel et al., 2013). Taking the impetus from studies within –often plant– ecology (Violle et al., 2014) and their successful application to understand the non-random distribution of animal and plant traits at regional (Ackerly and Cornwell, 2007) and global scales (Harfoot et al., 2014), trait-based models are increasingly utilized in microbial ecology to simplify our representation of complex communities and to parse out the mechanisms by which communities interact, function and emerge under different biotic and abiotic constraints. While we are aware of no studies that have applied a trait-based modeling approach specifically to understand the ecology of soil nitrite oxidizers, first attempts have been made to apply trait-based models to depict complex communities contributing to both phylogenetically narrow and broadly distributed processes (Follows et al., 2007; Allison, 2012; Bouskill et al., 2012; Ward et al., 2013). These models span multiple spatial scales from chemostat level (Bouskill et al., 2012), through mm to cm scale (Allison, 2012) all the way to global representation of communities (Follows et al., 2007). However, despite these recent progresses (see also Wallenstein and Hall, 2012), trait-based approaches are still in their infancy in microbial ecology and a number of challenges remain (Krause et al., 2014). In particular, the physiological traits important in determining fitness across environmental gradients are unknown for the vast majority of bacteria, and metabolic diversity, genomic plasticity and linkages between distinct traits often hinder the simple assignment of soil bacteria to distinct functional groups. In addition the testing of the trait-based model approach requires complementary model development and model output evaluation using laboratory or field experiments.

Herein, we report on the response to multifactorial global change treatments of soil nitrite-oxidizing bacteria, NOB, and the corresponding development of a trait-based model that links NOB ecophysiological traits with NOB fitness and rates of nitrite oxidation. Two major genera of NOB are present in soil, i.e., *Nitrobacter* and *Nitrospira* (Freitag et al., 2005; Spieck and Lipski, 2011; Pester et al., 2014), which play a key role in soil N dynamics by mediating nitrite oxidation, the second step of nitrification. Ammonia oxidation is often assumed to be the rate-limiting step of nitrification, but nitrite oxidation can limit the rate of nitrification in disturbed soil systems (Gelfand and Yakir, 2008; Roux-Michollet et al., 2008), thus influencing N availability to plants and N losses from ecosystems. Functional diversity exists within each of the two groups (e.g., Maixner et al., 2006), but *Nitrospira*-like NOB are assumed to have low half-saturation constants for nitrite, ${\mathrm{NO}}_{2}^{-}$, and oxygen, O_2_, and are favored by low availability of ${\mathrm{NO}}_{2}^{-}$ and O_2_, whereas *Nitrobacter*-like NOB have higher half-saturation constants and outcompete *Nitrospira* under conditions of high ${\mathrm{NO}}_{2}^{-}$ and O_2_ availabilities (Schramm et al., 2000; Wagner et al., 2002; Manser et al., 2005; Blackburne et al., 2007; Nowka et al., 2015). This has been supported by studies on NOB dynamics in chemostats and biofilm systems (Kim and Kim, 2006; Nogueira and Melo, 2006) and soils (Attard et al., 2010; Wertz et al., 2012). In addition, there is growing evidence that some *Nitrobacter* (Bock et al., 1983, 1990) and some *Nitrospira* (e.g., Daims et al., 2001; Gruber-Dorninger et al., 2015) are able to grow mixotrophically or heterotrophically as well. According to the current knowledge, adaptation of *Nitrobacter* and *Nitropira* to high and low N availability, respectively, is a main ecological difference between these two groups (Gruber-Dorninger et al., 2015) and mixotrophic capacity can be important in particular for some *Nitrobacter* (Degrange et al., 1997). This background on the ecology of NOB thus provides the rationale for distinguishing NOB groups in regard to their response to major environmental factors, i.e., nitrogen availability, organic carbon (OC) availability and O_2_ levels, making the NOB particularly amenable to trait-based modeling.

We tested the hypothesis that classifying soil NOB based on a few traits linked to their physiology and growth characteristics can allow us to identify the key controls over changes in the community composition and activity of these bacteria and predict their responses to a range of global change treatments (Supplementary Figure S1). We first characterized the responses of the potential activity, abundance and community composition of grassland soil NOB to multifactorial global change treatments, i.e., exposition to eight global change treatments through manipulation of three environmental drivers: atmospheric CO_2_ concentration, precipitation and N level, alone and in combination. We then demonstrated how a trait-based modeling approach can resolve an emergent NOB community structure consistent with the observations from different multifactorial global change treatments. We also evaluated the added value of the categorization of soil NOB into a few functional types, analyzing to what extent we need to represent a sufficient number of functional types in the trait-based model to adequately predict NOB responses to multifactorial global change.

## Materials and Methods

### Experimental Design and Soil Sampling

This study was conducted at the Jasper Ridge Global Change Experiment (JRGCE) in central California (37°24′N, 122°14′W, 150 m a.s.l.). The JRGCE was initiated in November 1998 in a moderately fertile grassland Mediterranean ecosystem, dominated by annual grasses and forbs. A detailed description of the experimental design and maintenance is given in Shaw et al. (2002) and Zavaleta et al. (2003). Here, we studied three global change manipulations – CO_2_, Precipitation and Nitrogen (N) – each at two levels (ambient and elevated). The elevated levels used for each main treatment were based on future global change scenarios for central California: (i) ambient and elevated atmospheric CO_2_, with the elevated CO_2_ treatment (680 μmol mol^-1^) manipulated with CO_2_ emitter rings; (ii) ambient (no addition) and elevated N deposition [7 g N-Ca(NO_3_)_2_ m^-2^ year^-1^]; (iii) ambient (no addition) and elevated precipitation (+50% over ambient plus an extension of the rainy season by 3 weeks with two additional rain events of 20 mm each) manipulated by a spray/drip irrigation system. It has been shown that over a long time scale (ca. 7 years after start of treatments), N fertilization with nitrate at Jasper Ridge induced a general increase of N cycling in soil and ultimately of gross ammonification through mineralization (Niboyet et al., 2011). The experiment provides a randomized block split-plot design including 12 plots (2 m diameter), each plot being divided into four 0.78 m^2^ quadrants. The four quadrants were separated belowground by fiberglass panels to a depth of 0.5 m, and aboveground by netting. Atmospheric CO_2_ was manipulated at the plot level while N addition (N) and precipitation (W) were manipulated at the quadrant level. Each treatment (referred to as CO_2_, N and W) and all their possible combinations (a total of eight treatments) were replicated six times, leading to a total of 48 subplots. Soil pH was 6.5–6.6 for all treatments.

Soil samples were collected at the time of peak plant biomass: on 26 April 2005 and on 27 April 2006 (i.e., at the end of the 7th and 8th growing seasons under treatments). At each date, one soil core from each of the 48 subplots was removed to a depth of 5 cm with a 5 cm diameter corer. Large roots and rocks were removed by hand and soil was homogenized. Gravimetric soil water content was determined by comparing the mass of a 5-g soil subsample before and after drying at 105°C. Assays for potential nitrite oxidation, PNO, were performed on fresh soil, whereas soil was stored at -20°C for DNA extraction to measure the abundance and diversity of NOB.

### Quantification of Gross Ammonification, Soil C Availability, and Potential Nitrite Oxidation

Gross ammonification, i.e., ammonification in the course of mineralization, was determined in April 2005 using ^15^N pool dilution (Niboyet et al., 2011). One 50-g soil sample from each quadrant was placed in a plastic bag and 3 mL of a ^15^N-(NH_4_)_2_SO_4_ solution was added (99 atom % ^15^N). The ^15^N label was homogenized with the soil by 15 min thorough mixing. A 10-g sub-sample was then taken and extracted with 25 mL 0.25 M K_2_SO_4_ for determination of the initial ${\mathrm{NH}}_{4}^{+}$ and ^15^N-${\mathrm{NH}}_{4}^{+}$ concentrations. The remaining soil samples were returned to the field in thin plastic bags, and buried in their original location. After a 24 h incubation period in the field, a second 10-g sub-sample was taken and extracted as above for determination of the final ${\mathrm{NH}}_{4}^{+}$ and ^15^N-${\mathrm{NH}}_{4}^{+}$ concentrations. Extracts were filtered and analyzed colorimetrically for ${\mathrm{NH}}_{4}^{+}$ concentrations using an autoanalyzer. ${\mathrm{NH}}_{4}^{+}$ and NO_3_^-^ were separated by diffusion, and ^15^N-${\mathrm{NH}}_{4}^{+}$> concentrations were determined using an elemental analyzer coupled to an isotope ratio mass spectrometer.

Soil CO_2_ production was measured in laboratory incubations in April 2005 and used as an indicator of soil OC availability (Brown et al., 2012). 15-g soil sub-samples were placed in 250-mL screw-top glass serum bottles and soil moisture was adjusted (0.21 g H_2_O g^-1^ dry soil). Bottles were sealed with screw caps lined with airtight Teflon-silicone septa and incubated for 48h in the dark at 25°C. Rates of CO_2_ production were calculated from three 15-mL headspace samples taken 30–60 min, 24 and 48 h after the incubation started and analyzed for CO_2_ concentrations on a gas chromatograph.

PNO data were obtained using the method described by Wertz et al. (2007) on the Microbial Activities in the Environment, AME, platform (Microbial Ecology Centre, Villeurbanne). Fresh soil samples (5 g equivalent dry mass) were incubated with 50 ml of NaNO_2_ solution (final concentrations: 50 μg N-${\mathrm{NO}}_{2}^{-}$ g^-1^ dry soil) at 28°C for 30 h with shaking (150 r.p.m.). PNO was measured as ${\mathrm{NO}}_{2}^{-}$ consumption rate during the nitrite-oxidation assays.

### Soil DNA Extraction and Measurement of NOB Abundances

DNA was extracted from frozen soil samples using the PowerSoil^TM^ DNA Isolation Kit (MO BIO Laboratories, Carlsbad, CA, USA).

*Nitrobacter*-like NOB abundance was determined by quantitative PCR on soil samples from April 2005 and April 2006. qPCR assays were carried out according to Attard et al. (2010). Tenfold standard serial dilutions ranging from 10^7^ to 10^1^ *nxrA* copies of genomic DNA from *Nitrobacter hamburgensis* X14 (DSMZ 10229) were used. Melting curves and amplified *nxrA* fragment length, observed after running an agarose gel for randomly chosen final PCR products, confirmed amplification specificity. To check for possible PCR inhibition, 10^5^ standard copies were co-amplified with 40 ng of soil DNA. Low inhibition of amplification (-5%) was observed, without any treatment effects.

*Nitrospira*-like NOB abundance was determined on soil samples from the two sampling dates. qPCR assays were carried out according to Attard et al. (2010). Tenfold standard serial dilutions ranging from 10^7^ to 10^1^ *Nitrospira* copies of linearized plasmid DNA (Accession number FJ529918) were used. Melting curves and amplified *16S* rRNA *Nitrospira* gene fragment length, observed after running an agarose gel for randomly chosen final PCR products, confirmed amplification specificity. We also checked the PCR efficiency for *Nitrospira* positive control, which was 95% in average.

### Characterization of the Major *Nitrobacter* Populations

The major *Nitrobacter*-like NOB populations were characterized by cloning and sequencing on all soil samples from April 2005. *Nitrobacter*-like *nxrA* genes were PCR amplified using 40 ng DNA and the same primers as for the qPCR approach described above. The PCR products were purified using the NucleoSpin^®^ Extract II kit (Macherey-Nagel, Düren, Germany) and were cloned using the pGEM T-Easy vector system (Promega Ltd, Southampton, UK) and JM109 supercompetent *Escherichia coli* cells (Stratagene Inc., Maidstone, UK). For each of the eight treatments, 3–5 clone libraries were constructed from 3 to 5 (out of the six) randomly selected replicates. From each clone library, 30 clones were sequenced (LGC Genomics, Berlin, Germany). Phylogenetic relationships were inferred by maximum-parsimony with the GTR-GAMMA model of substitution using RAxML, and clusters of close relative sequences were built at a patristic distance of 0.03 substitutions per nucleotide using RAMI, considering reference sequences previously published and four outgroup narG sequences. The robustness of cluster identification was tested using the Unifrac Significance after 1000 permutations and clusters showing a *p*-value < 0.001with all other clusters were further considered for analysis. Sequences have been submitted to GenBank (accession numbers: KJ021461- KJ021624).

### Trait-Based Modeling Approach

The trait-based model was adapted from Bouskill et al. (2012) and NOB functional trait values (enzymatic kinetics of nitrite oxidation including half-saturation constant for substrate, use of OC, and response to O_2_) were derived from literature surveys (Supplementary Table S1). In the reference runs, the model represents three coherent groups of NOB: K-strategists for ${\mathrm{NO}}_{2}^{-}$ (*Nitrospira* spp.) irrespective of their possible mixotrophic capacity; r-strategists for ${\mathrm{NO}}_{2}^{-}$ that perform best as chemolitotrophs and essentially assimilate CO_2_ (i.e., *Nitrobacter* spp. without or with low mixotrophic capacity); and r-strategists for ${\mathrm{NO}}_{2}^{-}$ that perform best as mixotrophs and substantially use OC (i.e., *Nitrobacter* like the strain *N. hamburgensis)*. The three bacterial groups are represented by functional traits related to enzyme kinetics, growth rates and growth efficiencies (increase in C-biomass given as a function of the number of moles of ${\mathrm{NO}}_{2}^{-}$ oxidized). NOB biomass, B, is represented by total biomass, biomass C and N, and cellular quotas for both (Bouskill et al., 2012). Equations governing the rate of cell division, substrate uptake and competitive dynamics have been described previously (Bouskill et al., 2012). Briefly, NOB acquire energy by oxidizing their substrate S: ${\mathrm{NO}}_{2}^{-}$ for *Nitrobacter* performing best as chemolithotrophs and *Nitrospira*, and either ${\mathrm{NO}}_{2}^{-}$ or OC for NOB performing best as mixotrophs, according to dual Monod kinetics:

(1) $$V_{NOB}^{S}=V_{MAX}^{S}\frac{\left[S\right]}{K_{M}^{S}+[S]}\frac{\left[O_{2}\right]}{K_{M}^{O_{2}}+[O_{2}]}B$$

where $V_{NOB}^{S}$ is the overall ${\mathrm{NO}}_{2}^{-}$ or OC oxidation rate (μM s^-1^), $V_{MAX}^{S}$ is the maximum oxidation rate [μM (M biomass)^-1^ s^-1^], $K_{M}^{S}$ and $K_{M}^{O_{2}}$ are the half saturation constants for ${\mathrm{NO}}_{2}^{-}$ or OC and for O_2_ (μM), respectively, and [S] and [O_2_] are ${\mathrm{NO}}_{2}^{-}$ or OC and O_2_ concentrations in the soil solution (μM), respectively. NOB fix carbon substrate C (CO_2_ for *Nitrobacter* performing best as chemolithotrophs and for *Nitrospira*, and CO_2_ or OC for NOB performing best as mixotrophs) following Michaelis–Menten kinetics:

(2) $$V^{C}=V_{MAX}^{C}\frac{\left[C\right]}{K_{M}^{C}+[C]}B$$

where $V_{MAX}^{C}$ is the maximal assimilation rate of C [μM (M biomass)^-1^ s^-1^], $K_{M}^{C}$ is the half-saturation constant for C (μM), and [C] is the C concentration in the soil solution (μM). $V_{MAX}^{C}$ depends on the maximal energy yielded by ${\mathrm{NO}}_{2}^{-}$ /OC oxidation and is thus related to $V_{MAX}^{NO_{2}^{-}}$ and to the efficiency of C fixed relative to ${\mathrm{NO}}_{2}^{-}$ /OC oxidized. Following assimilation, biomass development is regulated by NOB growth yield and the energy generated, and the C-requirement of the cell to satisfy growth terms (Bouskill et al., 2012). Changes in biomass over time are calculated as:

(3) dBdt =μMAXimin{di}Bi−ΔBi

where $\mu_{MAX}^{i}$ represents the maximum specific growth rate (s^-1^) of the *i^th^* NOB group, *d_i_* is the C:N quota governing growth rate, and Δ represents the first order microbial mortality rate (s^-1^) (Bouskill et al., 2012).

In contrast to the previously described model, the current code resolved three main soil NOB functional types, trait values being derived from an extensive literature survey (Supplementary Table S1). The new parameterization takes the metabolic flexibility of some NOB into account: in particular, values for μ_max_ and K_M_(O_2_) for NOB performing best as mixotrophs change as a function of OC concentrations. The μ_max_ values of these NOB vary from 6.9 × 10^-6^ s^-1^ without OC to 27.8 × 10^-6^ s^-1^ when OC concentration is 150 ppm, whereas their K_M_(O_2_) values range from 25.1 μM at low O_2_ tension (∼ 3.2 ppm) up to 125 μM under high O_2_ supply (>200 ppm) (Bock et al., 1983; Laanbroek et al., 1994).

We sought to explore the niches of the three NOB groups by examining their biomass and activity across substrate gradients (${\mathrm{NO}}_{2}^{-}$ and OC) and O_2_. The model was run to steady state under constant concentrations of ${\mathrm{NO}}_{2}^{-}$, OC (as CH_2_O) and O_2_. Simulated ${\mathrm{NO}}_{2}^{-}$ concentrations ranged from 7.4 × 10^-6^ to 3.7 × 10^-2^ M to cover the likely range of concentrations experienced in soils and under culture conditions. The simulated OC concentrations covered a gradient of 0–5 × 10^-3^ M. Simulated O_2_ concentrations represented a low (O_2_ = 1 × 10^-4^ M) to high (O_2_ = 1 × 10^-2^ M) gradient, and signified the likely O_2_ gradients associated with conditions of high respiration activity or high moisture (low O_2_) or atmospheric intrusion (high O_2_).

Since the batch model did not represent explicitly spatial gradients of environmental conditions, we computed NOB dynamics for two homogeneous soil compartments. The first compartment corresponded to a bulk of soil microhabitats exposed to highest O_2_ and nitrogen availabilities, which harbor *Nitrobacter* in addition to *Nitrospira.* This compartment was set to 5.5% of soil mass according to Grundmann et al. (2001). Resource availability in this compartment varied with ammonification and respiration rates which were affected by treatments. Since gross ammonification was found to be a good proxy of N availability to NOB (Attard et al., 2010), changes in ${\mathrm{NO}}_{2}^{-}$ concentration (M) were related to changes in gross ammonification (ng-N g^-1^ h^-1^): NO_2_ = 2 × 10^-16^ (ammonification)^5.35^. Given that soil respiration rate measured under lab conditions without OC supply is a proxy of native soil OC availability (Brown et al., 2012), changes in actual soil OC availability (M) among treatments were estimated from changes in respiration (mg-C g^-1^ h^-1^) : OC = 10 ^∗^ Respiration -15.5. Based on concurrent measurements of soil respiration and O_2_ concentration in soil atmosphere for different treatments at the Jasper Ridge grassland site, changes in O_2_ concentration (M) were negatively related to changes in respiration (mg-C g^-1^ h^-1^): O_2_ = -0.019 ^∗^ ln(Respiration) + 0.0137. The second soil compartment represented a bulk of other habitats and was suitable at best for *Nitrospira*, with O_2_ and ${\mathrm{NO}}_{2}^{-}$ availabilities prescribed at low values (5 × 10^-4^M and 10^-5^M, respectively).

While reference model runs included the three NOB functional types as defined in Supplementary Table S1, we also performed three additional sets of runs. The first additional runs represented only two NOB types defined according to genus, i.e., *Nitrospira* (same traits as in Supplementary Table S1) and an ‘average’ *Nitrobacter* type with functional traits that were computed as means of traits of the two *Nitrobacter* types (including weakly flexible response to OC). The second additional runs represented only one ‘average’ NOB type, i.e., with functional traits computed as means of traits of the three types used in the reference runs. The third additional runs represented four NOB types, i.e., distinguishing *Nitrospira* performing best as chemolitotrophs and *Nitrospira* performing best as mixotrophs in addition to the two *Nitrobacter* functional types (trait values provided in Supplementary Table S1).

### Statistical Analyses

Nitrite-oxidizing bacteria abundance and PNO data were analyzed for each sampling date with PROC MIXED in SAS 9.3 (SAS Institute, Cary, NC, USA) using a three-way split-plot analysis of variance that included: (i) CO_2_ as a fixed whole-plot effect; and (ii) N and W as fixed split-plot effects; and (iii) interaction terms for all possible treatment combinations. PNO and NOB abundance data were log-transformed to correct non-homogeneity of variance. Effects with *p* < 0.05 are referred to as significant, and effects with *p* ranging from 0.05 to 0.10 as marginally significant. Overall main treatment effect sizes were calculated as follows: % effect = 100% ^∗^ [elevated – ambient]/ambient. The effect of treatments on the percentage of *Nitrobacter*-like NOB belonging to key clusters was tested by analysis of variance and means comparison using Fisher’s test.

The treatment effects on the whole community structure of *Nitrobacter*-like NOB were analyzed with the PRIMER software (vs. 6, PRIMER-E Ltd, Plymouth, UK) using non-metric multidimensional scaling (MDS) and one-way analysis of similarities (ANOSIM) to compare the genetic structures among each pair of global change treatments.

The correlations between the predicted and observed relative abundances of each NOB functional type and between predicted actual nitrite oxidation rate and observed PNO were assessed by linear or non linear regressions.

## Results

### Multifactorial Global Change Effects on NOB Community Structure, Abundance, and Activity

**FIGURE 1:**
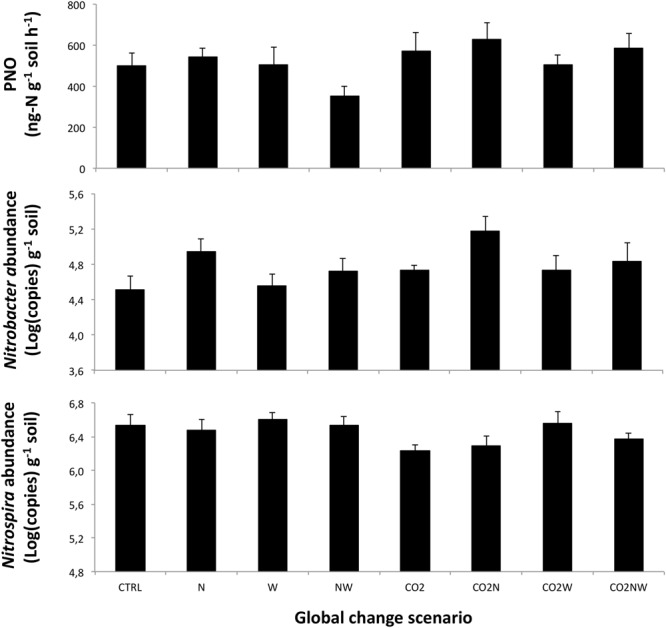
**Observed responses of (Top) potential nitrite oxidation, PNO, (Middle) the abundance of total *Nitrobacter*-like NOB, and (Bottom) the abundance of *Nitrospira* in response to the different global change scenarios in April 2005.** Means are presented with standard errors (*n* = 6). N, W and CO_2_ refer to nitrogen addition, increased precipitation, and elevated atmospheric CO_2_, respectively. CTRL refers to the control treatment where all factors are at ambient levels. Results of statistical analyses of treatment effects are presented in **Table 1**.

We observed significant effects of the multifactorial global change treatments on the community structure, abundance and potential activity of NOB. When considering main effects across all treatments, soil potential nitrite oxidation rate was enhanced by elevated CO_2_ (+25 and +56% 7 and 8 years after the initiation of treatments, respectively), reduced by elevated precipitation (-16 and -17%), and unaffected by N addition (**Table 1**, **Figure 1**; Supplementary Figure S2). At both sampling dates, the abundance of *Nitrobacter* tended to increase under elevated CO_2_, decreased under elevated precipitation (-40 and -35%), and increased under high N (+93 and +67%) (**Table 1**, **Figure 1**; Supplementary Figure S3). In contrast, the amplitude of the response of *Nitrospira* abundance to main global change treatments was generally lower than that of *Nitrobacter* (**Table 1**, **Figure 1**; Supplementary Figure S3). N effect on *Nitrospira* abundance was weak and not consistent between both years, while CO_2_ and increased precipitation tended to decrease and increase, respectively, *Nitrospira* abundance (i.e., opposite effects than those observed on *Nitrobacter* abundance). The abundance of *Nitrobacter* and *Nitrospira* actually increased and slightly decreased, respectively, with increased gross ammonification (Supplementary Figure S4).

**Table 1:** *p*-values from three-way split-plot analysis of variance testing for the effects of global change scenarios on potential nitrite oxidation and abundance of *Nitrobacter*-like NOB and *Nitrospira* at the end of the 7th and 8th plant growing seasons under treatments (April 2005 and April 2006, respectively).

	Potential nitrite oxidation		*Nitrobacter* abundance		*Nitrospira* abundance	
Treatment	% Effect	*p*-value	% Effect	*p*-value	% Effect	*p*-value
**April 2005**
CO2	**+25**	**0.06**	+94	0.11	**-37**	**0.06**
W	**-16**	**0.01**	**-40**	**0.06**	**+50**	**0.06**
N	**-**3	0.58	**+93**	**0.03**	**-**3	0.65
CO2^∗^W		0.39		0.62		0.62
CO2^∗^N		0.17		0.83		0.72
W^∗^N		0.29		0.14		0.66
CO2^∗^W^∗^N		**0.04**		0.63		0.28
**April 2006**
CO2	**+56**	**0.005**	+74	0.11	**-**11	0.82
W	**-17**	**0.03**	**-35**	**0.04**	+28	0.24
N	**-**2	0.99	+67	0.80	**+22**	**0.03**
CO^∗^W		0.72		0.92		0.94
CO^∗^N		0.53		0.96		0.82
W^∗^N		0.37		0.34		0.51
CO^∗^W^∗^N		0.74		0.10		0.37

Treatments are elevated CO_2_ (CO2), increased precipitation (W), N addition (N), and all their combinations, i.e., eight treatments (*n* = 6 per treatment). Effects of each main treatment were calculated as: % effect = 100 * [elevated – ambient]/ambient (*n* = 24 in the elevated treatment and *n* = 24 in the ambient treatment). Significant effects (*p* < 0.05) and marginally significant effects (*p* ranging from 0.05 to 0.1) are indicated in bold.

**FIGURE 2:**
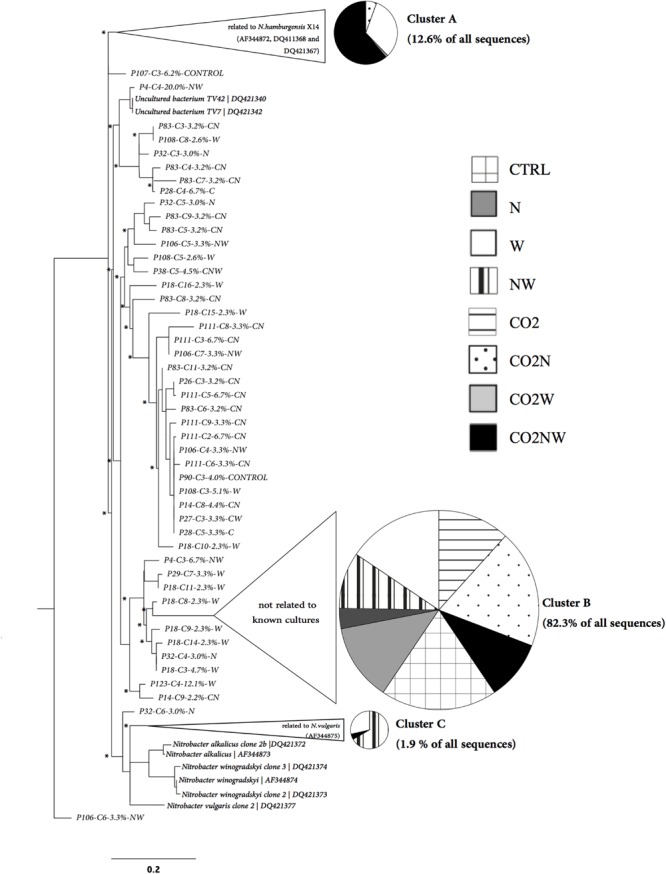
**Phylogenetic tree of *Nitrobacter*-like *nxrA* sequences retrieved from soils experiencing different global change treatments in relation to *nxrA* sequences of cultivated strains.** Disks represent the distribution of *nxrA* sequences among treatments for the three main clusters identified (Unifrac Significance for each: *p* < 0.001). These three clusters correspond to 96.8% of all the *nxrA* sequences retrieved across treatments and to more than 86% of *nxrA* sequences retrieved for each treatment.

Community structure analyses were focused on *Nitrobacter* because sensitivity tests with the trait-based model demonstrated that its abundance drives changes in nitrite oxidation in the studied soils. The *Nitrobacter* community structure differed significantly among global change treatments (Supplementary Table S2). In particular, community compositions were similar under combined elevated CO_2_, nitrogen addition and elevated precipitation (CO_2_NW) and under elevated precipitation (W), but differed from those under other global change treatments (Supplementary Table S2). Phylogenetic analysis of *Nitrobacter*-like NOB demonstrated selection of specific populations under certain treatments (**Figure 2**). In particular, *Nitrobacter* populations related to the cultivated strain *N. hamburgensis*, rare in control soil, became dominant (63% of the community) under CO_2_NW treatment and, to a lesser extent (31% of the community), under W treatment (**Figure 2**; **Table 2**), and their abundance increased with increased OC availability in soil (Supplementary Figure S5). The mechanisms that underlie the observed responses of NOB to global change treatments were, however, difficult to discern from these data alone, and trait-based modeling was used to better understand and predict the observed responses.

**Table 2:** Variations in the percentage of *Nitrobacter*-like NOB distributed according to global change treatments among the three main NOB clusters identified in **Figure 2**.

Treatments	Cluster A	±SE	Cluster B	±SE	Cluster C	±SE
CTRL	0.0^a^	±0.0	98.0^a^	±1.3	0.0^a^	±0.0
N	0.0^a^	±0.0	96.0^a^	±4.0	0.0^a^	±0.0
W	30.8^b^	±11.4	52.6 ^bc^	±12.0	4.2^a^	±2.9
NW	0.0^a^	±0.0	71.1^ab^	±18.5	15.6^b^	±11.0
CO2	0.0^a^	±0.0	96.7^a^	±3.3	0.0^a^	±0.0
CO2N	4.2^a^	±3.4	82.7^a^	±6.6	0.0^a^	±0.0
CO2W	0.9^a^	±0.9	98.1^a^	±1.0	0.0^a^	±0.0
CO2NW	62.8^c^	±14.5	34.7^c^	±14.8	1.0^a^	±1.0


Mean values are presented with the corresponding standard errors. Treatment effect was significant for *Nitrobacter* from cluster A affiliated to *N. hamburgensis* (*p* < 0.0001) and for *Nitrobacter* from cluster B not affiliated to any cultivated strain (*p* = 0.0003), and marginally significant for cluster C (*p* = 0.06). For each cluster, different letters indicate significant difference between treatments (*p* < 0.05).

### Reconstructed Ecological Niches of the 3 NOB Groups by Trait-Based Modeling

**FIGURE 3:**
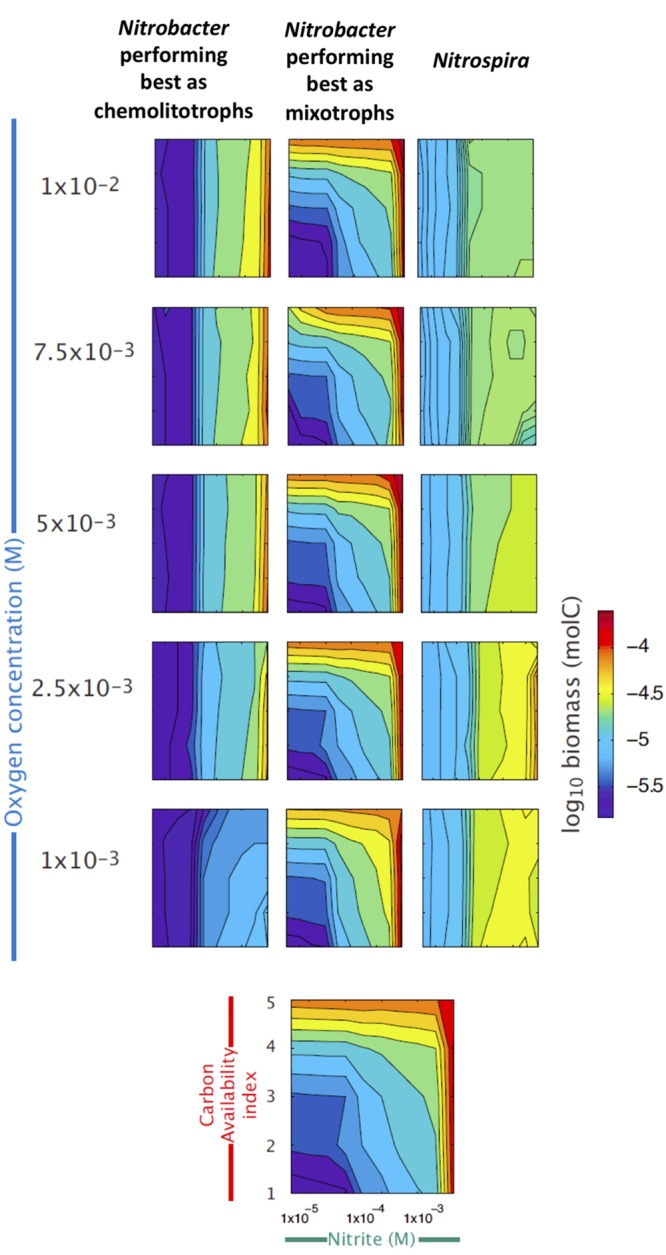
**Reconstructed niches of the three NOB groups across environmental gradients.** Simulated changes in the abundances of the three functional types of nitrite oxidizers (see Supplementary Table S1), expressed as log_10_ of biomass, are presented across gradients of nitrite (*X* axis), organic carbon (red *Y* axis), and oxygen availabilities (blue *Y* axis). The last panel at the bottom provides the scales used in all panels.

Using the trait-based model and the known ecophysiological traits of these organisms, we reconstructed the niche of each of the three groups across gradients of ${\mathrm{NO}}_{2}^{-}$, OC and O_2_ concentrations (**Figure 3**). Simulation results confirm that *Nitrospira* perform well under low O_2_ and relatively low ${\mathrm{NO}}_{2}^{-}$ availabilities, whereas mainly chemolitotrophic *Nitrobacter* perform well under high ${\mathrm{NO}}_{2}^{-}$ and O_2_ concentrations (**Figure 3**). Although NOB performing best as mixotrophs can simultaneously use ${\mathrm{NO}}_{2}^{-}$ and OC, simulations results show that they tend to be outcompeted by the two other NOB groups under low ${\mathrm{NO}}_{2}^{-}$ concentrations, while their contribution to NOB community and nitrite oxidation increases with increasing OC and ${\mathrm{NO}}_{2}^{-}$ concentrations.

### Predicted Responses of Soil NOB to Multifactorial Global Change

**FIGURE 4:**
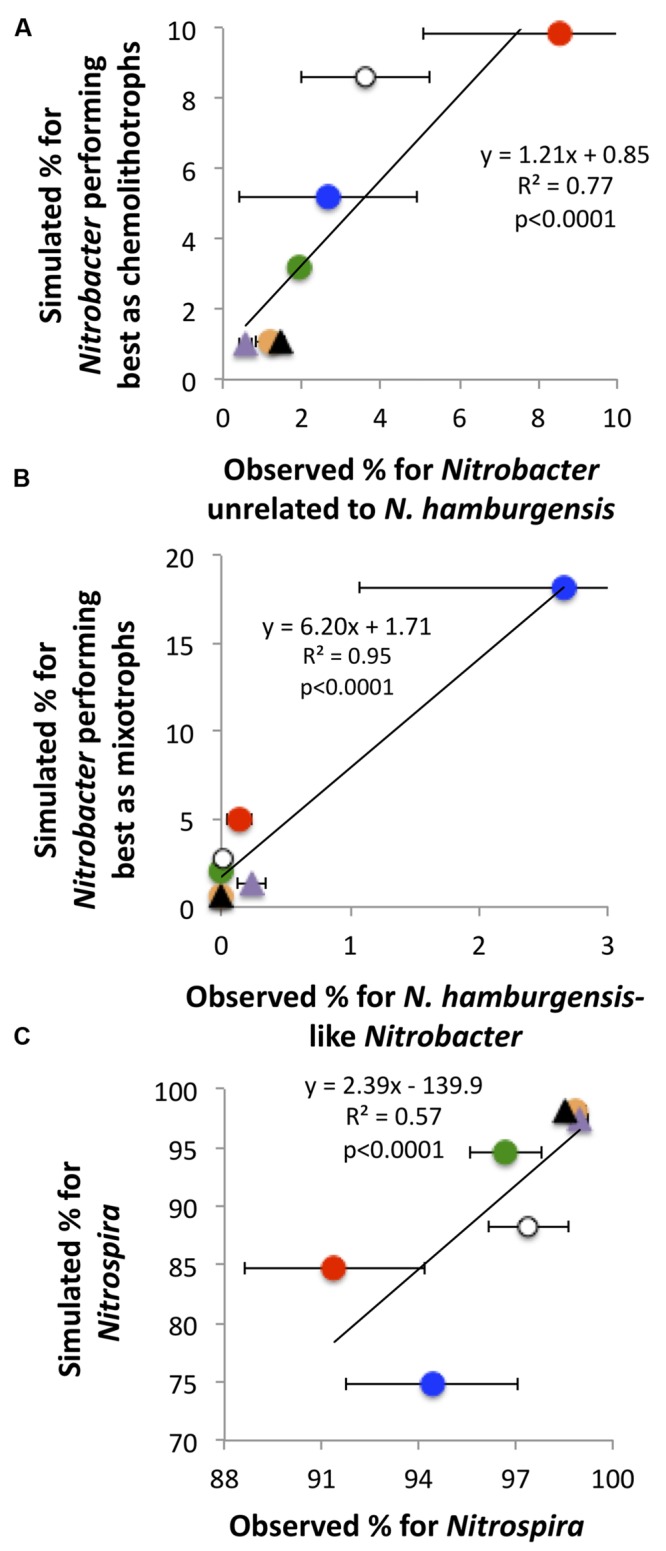
**Observed and predicted relative contributions of the three bacterial functional types to the total soil nitrite-oxidizing community (A: chemolithotrophic *Nitrobacter*; B: mixotrophic *Nitrobacter*; C: *Nitrospira*).** Observations (*X*-axis) are mean values with standard errors for each global change treatment. Orange circle, CTRL; purple triangle, W; black triangle, NW; green circle, CO_2_; red circle, CO_2_N; white circle, CO_2_W; blue circle, CO_2_NW. Modeling was not possible for the N treatment due to lack of measurements of N availability in soil. For **(B)**, simulation is for all mixotrophic *Nitrobacter*, whereas observation is for *Nitrobacter* affiliated to the strain *N. hamburgensis* disregarding the possible presence of other mixotrophic populations not affiliated to cultivated strains.

The simulated NOB community captured reasonably well the observations of NOB communities in the field (**Figure 4**). For instance, the predicted values of the relative abundance of *Nitrobacter* performing best as mixotrophs were highest under the CO_2_NW treatment, corresponding to high substrate availability (OC + ${\mathrm{NO}}_{2}^{-}$) and relatively low O_2_ availability, which is consistent with the highest values observed for *Nitrobacter* affiliated to *N. hamburgensis* which putatively have substantial mixotrophic capacity. Simulated and observed relative abundances of *Nitrospira* were also significantly correlated, and both were lowest under the CO_2_N and CO_2_NW treatments and highest under the treatments characterized by low N availability (**Figure 4C**).

**FIGURE 5:**
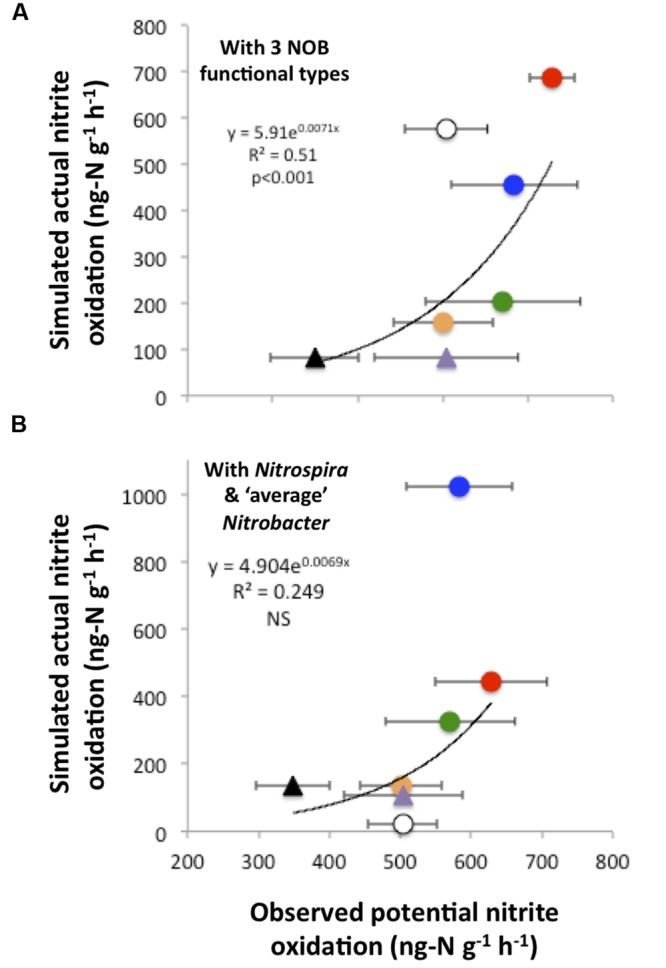
**Simulated actual nitrite oxidation compared to observed potential nitrite oxidation.** The relationship is presented when using the trait-based model with three functional types of nitrite-oxidizing bacteria **(A)**. For comparison, results are presented when disregarding the functional diversity within *Nitrobacter*, i.e., considering only *Nitrospira* and ‘average’ *Nitrobacter* **(B)**. Each point corresponds to one global change scenario, and observed mean values are presented with standard errors. Symbols are as in **Figure 4**.

In addition, there was a significant relationship between simulated actual nitrite oxidation and observed PNO, which were at their highest and their lowest under the CO_2_N and NW treatments, respectively (**Figure 5A**). Additional simulations that did not account for the functional diversity within the *Nitrobacter* (i.e., representing the *Nitrobacter* as one ‘average’ type) poorly reflected observed rates of nitrite oxidation (**Figure 5B**). In particular, predicted nitrite oxidation rate was too high under CO_2_NW, with the ‘average’ *Nitrobacter* type outcompeting *Nitrospira* as a result of its combination of moderate half-saturation constants for ${\mathrm{NO}}_{2}^{-}$ and O_2_ and relatively high maximal oxidation and growth rate. With no functional diversity within NOB (i.e., representing one NOB type with ‘average’ trait values), the model predicted much too low rates of nitrite oxidation (<20 ng-N g^-1^ h^-1^) under CTRL, W and NW treatments that are characterized by low ${\mathrm{NO}}_{2}^{-}$ and OC availability (not shown). Thus, the modeled ‘average’ NOB did not perform well at low resource levels, whereas some NOB are actually able to maintain appreciable rates of nitrite oxidation as observed in the field. In contrast, the additional runs that represented four NOB types, i.e., distinguishing *Nitrospira* performing best as chemolitotrophs and *Nitrospira* performing best as mixotrophs in addition to the two *Nitrobacter* functional types, yielded results very similar to the control runs, both in terms of NOB community composition (Supplementary Figure S6) and nitrite oxidizing activity (Supplementary Figure S7).

## Discussion

Soil microorganisms play an important role in the responses of terrestrial ecosystems to global change and feedback to climate (Singh et al., 2010), but responses of the soil microbiota to global change has so far been largely neglected in terrestrial ecosystem and biogeochemical cycling models. Tackling this issue requires (i) to quantify the responses of key soil microbial functional groups to multifactorial global change, including possible interaction effects between different global change factors; and (ii) to identify key environmental drivers and ecological niche characteristics of the microbial groups studied, and develops approaches to predict the responses to multifactorial global change.

Here, we demonstrated that elevated CO_2_, increased precipitation and nitrogen addition, alone or in combination, altered the abundance, community structure and activity of soil nitrite oxidizing bacteria. Contrasted responses to global change treatments were observed between three groups of NOB, i.e., *Nitrobacter* related to *N. hamburgensis, Nitrobacter* unrelated to *N. hamburgensis*, and *Nitrospira*, which could be related to their key ecophysiological traits as inferred according to our current knowledge. Indeed, the observed decrease in *Nitrospira* abundance and increase in *Nitrobacter* abundance with increasing N availability in soil are consistent with previous studies reporting that *Nitrospira*-like NOB have low half-saturation constants for ${\mathrm{NO}}_{2}^{-}$ and O_2_, whereas *Nitrobacter*-like NOB perform better under conditions of high ${\mathrm{NO}}_{2}^{-}$ and O_2_ availabilities (Schramm et al., 2000; Wagner et al., 2002). Our results are also consistent with the fact that some *Nitrobacter*, like *N. hamburgensis*, can grow better mixotrophically using both ${\mathrm{NO}}_{2}^{-}$ and OC and perform well under relatively low O_2_ supply (Bock et al., 1983). Indeed, putatively mixotrophic *Nitrobacter* affiliated to *N. hamburgensis*, rare under most treatments, became dominant under the ‘High CO_2_ + Nitrogen + Precipitation’ treatment that was characterized by high OC and ${\mathrm{NO}}_{2}^{-}$ availability. However, unraveling the precise mechanisms and environmental drivers explaining the global change effects on NOB was hardly tractable because different soil environmental drivers co-varied in response to global change treatments, and the global change effects were hardly predictable only based on observations.

Our work shows that applying a trait-based modeling approach categorizing NOB functional types according to key traits allows identification of the ecophysiological and environmental drivers of the observed changes in community composition and prediction of the responses of NOB to multifactorial global change. First, the simulation results, including reconstructed niches of each NOB type, clearly demonstrate that change in N availability is the main driver of the *Nitrobacter*-to-*Nitrospira* ratio in soil. The model outputs also show that the overall taxonomic changes in the soil NOB communities remain relatively low across all treatments – and more generally across a broad range of environmental conditions – since soil NOB communities were quantitatively always dominated by *Nitrospira* in Jasper Ridge soils, as already reported for other soils (Attard et al., 2010; Wertz et al., 2012). But the changes were large from a functional point of view, which highlights that *Nitrobacter* groups play very important functional roles despite their low abundances in soil as previously suggested (Attard et al., 2010). As supported by our modeling results, the explanation is that the specific activity is much lower for *Nitrospira* than *Nitrobacter*, and *Nitrospira* are preferentially located in microhabitats characterized by low resource availability where they are not outcompeted by *Nitrobacter* but where their activity is particularly low. Second, the modeling approach demonstrates that a marked increase in the availability of OC and ${\mathrm{NO}}_{2}^{-}$, in conjunction with relatively low O_2_ tension, was the key driver of the emergence of *Nitrobacter* performing well as mixotrophs. However, the simulated relative abundance of these *Nitrobacter* was higher than that of NOB related to *N. hamburgensis* (**Figure 4B**). This suggests either a bias in simulation outputs or that there are additional *Nitrobacter* with high mixotrophic capacity in soil beyond those related to known, cultivated strains. Third, the trait-based approach resolved an emergent NOB community structure broadly consistent with the observations from the different treatments, but distinguishing at least three functional groups was needed to predict the response of NOB and their activity to the global change scenarios. Representing one or two groups did not allow adequate simulations of NOB response, while representing four groups did not yield better simulated responses. The trait-based modeling approach can thus guide the level of complexity and diversity (i.e., number of functional types and traits represented) that is required when representing microbial functional groups in terrestrial ecosystem models.

Our predictions of soil NOB responses to future global change have important implications with respect to the N cycle. In particular, the emergence of NOB performing best as mixotrophs as dominant members of the community under the CO_2_NW treatment could lead to a novel, direct coupling between the second step of nitrification and organic C availability and an altered balance between ammonification and nitrification. Since mixotrophic capacity is also likely more important than anticipated for ammonia oxidizers (Prosser and Nicol, 2012), mixotrophic nitrifiers may explain why some high-carbon ecosystems exhibit appreciable rates of nitrification (Stark and Hart, 1997) and challenge the notion that carbon inputs to soil should restrict nitrification by stimulating microbial immobilization (Hungate et al., 1997). Furthermore, a direct coupling between nitrification and the C cycle beyond CO_2_ assimilation suggests potential new controls over ecosystem production and consumption of nitrogen trace gases and regulation of nitrogen availability to plants. This should be further explored, including for agricultural land experiencing organic C inputs.

Reliably generalizing trait-based approaches in microbial ecology will depend on our knowledge of bacterial ecophysiological traits (Fierer et al., 2007), trade-offs between traits (Schuetz et al., 2012; Phan and Ferenci, 2013), and the coupling between trait and phylogenetic affiliation (Salles et al., 2012). Here, the broad congruency between NOB phylogeny and functional traits made the trait-based approach successful. Feasibility of trait-based modeling needs to be systematically explored for other bacterial groups (Philippot et al., 2010; Salles et al., 2012), but the overall importance of vertical inheritance for the phylogenetic dispersion of functional traits – in particular complex traits – in microorganisms (Martiny et al., 2013) paves the way for successful use of trait-based modeling in microbial ecology.

Our study demonstrates that coupling field observations with trait-based modeling facilitates understanding the biological and environmental controls on changes in soil bacterial community composition and function, and predicting soil bacterial response to environmental change. In the future, soil microbial diversity may be represented in ecosystem models as microbial functional types, similar to the approach successfully used for plant communities (Poulter et al., 2011).

## Author Contributions

XLR designed and led the study. The modeling approach was led by NB in close link with XLR and with inputs by JT. The long term field experiment was led by CF. Field sampling and measurements of microbial activities and environmental variables were made by AN, LB, PD, BH, XLR, and FP. Molecular analyses were made by FP, TP, XLR, MT, CL, and AT. XLR wrote the paper, with major inputs from NB, BH, AN, and CF. All authors discussed the results and commented on the manuscript.

## Conflict of Interest Statement

The authors declare that the research was conducted in the absence of any commercial or financial relationships that could be construed as a potential conflict of interest.
