# Supplementary material for: Predicting the Responses of Soil Nitrite-Oxidizers to Multi-Factorial Global Change: A Trait-Based Approach
Source: Front Microbiol. 2016 May 17;7:628. doi: 10.3389/fmicb.2016.00628 (PMC4868854; doi:10.3389/fmicb.2016.00628)

Front. Microbiol. 7:628. doi: 10.3389/fmicb.2016.00628

Supplementary Material

Predicting soil bacterial responses to multi-factor global change with trait-based modeling

Xavier Le Roux*, Nicholas J. Bouskill, Audrey Niboyet, Laure Barthes, Paul Dijkstra, Chris B. Field, Bruce A. Hungate, Catherine Lerondelle, Thomas Pommier, Jinyun Tang, Akihiko Terada, Maria Tourna & Franck Poly

*** Correspondence:** xavier.le-roux@univ-lyon1.fr

SUPPORTING INFORMATION:

Supplementary Tables 1 & 2

Supplementary Figures 1, 2, 3, 4, 5, 6 & 7

SUPPLEMENTARY TABLES

Supplementary Table S1. Functional trait values of the different groups of nitrite-oxidizing bacteria, NOB, represented in the modeling approach. The range of reported values is indicated, while mean values were used to run simulations. Key references used to determine trait values for each group are provided below.

Reference simulations using 3 NOB groups

**Traits** Functional group of soil NOB	**Use of C_{org}**	**V_{max}^{NO2} (µM M biomass-C^{-1} s^{-1})**	**K_M^{NO2} (µM)**	**μ_{max} (10^{-6} s^{-1})**	**K_M^{O2} (µM)**	**References**
Nitrobacter performing best as chemolitotrophs	- (+)	2331-9136	49-606	5.56-15	22-256	See below
Nitrobacter performing best as mixotrophs	+	5432-12840	706-1370	6.94-27.8 (C-dependent)	25.1-135 (O_2-dependent)	See below
Nitrospira	-/+	49.4-262	9-29	3.01-3.82	4.1-16.9	See below

V_{max}^{NO2} : maximal nitrite oxidation rate; K_M^{NO2} : half saturation constant for nitrite; μ_{max} : maximum growth rate; K_M O_2 : half saturation constant for O_2; K_M^{Corg} a was set to 10mM (no value found in the literature) ; all other model parameters as in (25)

Sensitivity analysis - simulations using 4 NOB groups

**Traits** Functional group of soil NOB	**Use of C_{org}**	**V_{max}^{NO2} (µM M biomass-C^{-1} s^{-1})**	**K_M^{NO2} (µM)**	**μ_{max} (10^{-6} s^{-1})**	**K_M^{O2} (µM)**	**References**
Nitrobacter performing best as chemolitotrophs	- (+)	2331-9136	49-606	5.56-15	22-256	See below
Nitrobacter performing best as mixotrophs	+	5432-12840	706-1370	6.94-27.8 (C-dependent)	25.1-135 (O_2-dependent)	See below
Nitrospira performing best as chemolitotrophs	- (+)	49.4	9	3.01	4.1	See below
Nitrospira performing best as mixotrophs	+	262	29	3.82	16.9	See below

References for *Nitrobacter* performing best as chemolitotrophs:

Both, G. J., Gerards, S. & Laanbroek, H. J. (1992). Kinetics of nitrite oxidation in two *Nitrobacter* species grown in nitrite-limited chemostats. *Arch. Microbiol.* 157: 436-441.

Gay, G. & Corman, A. (1984). Comparative study of the growth of two strains of *Nitrobacter* in batch and continuous culture. *Microbial Ecol.* 10: 99-105.

Hunik, J. H., Meijer, H. J. G. & Tramper, J. (1993). Kinetics of *Nitrobacter agilis* at extreme substrate, product and salt concentrations. *Appl. Microbiol. Biotechnol*. 40: 442-448.

Josserand, A., Gay, G. & Faurie, G. (1981). Ecological study of two *Nitrobacter* serotypes coexisting in the same soil. *Microbial Ecol.* 7: 275-280.

Keen, G. A. & Prosser, J. I. (1987). Steady state and transient growth of autotrophic nitrifying bacteria. *Arch. Microbiol.* 147: 73- 79.

Kim, D. J. & Kim, S. H. (2006). Effect of nitrite concentration on the distribution and competition of nitrite-oxidizing bacteria in nitratation reactor systems and their kinetic characteristics. *Water Res.* 40: 887–894.

Laanbroek, H. J. & Gerards, S. (1993). Competition for limiting amounts of oxygen between *Nitrosomonas europaea* and *Nitrobacter winogradskyi* grown in mixed continuous cultures. *Arch. Microbiol.* 159: 453-459.

Prosser, J. I. (1989). Autotrophic nitrification in bacteria. *Adv. Microbiol. Physiol.* 30: 125-181.

Underhill, S. E. & Prosser, J. I. (1987). Surface attachment of nitrifying bacteria and their inhibition by potassium ethyl xanthate. *Microbial Ecol.* 14: 129-139.

References for *Nitrobacter* performing best as mixotrophs:

Bock, E., Sundermeyer-Klinger, H. & Stackebrandt, E. (1983). New facultative lithoautotrophic nitrite-oxidizing bacteria. *Arch. Microbiol.* 136: 281–284.

Both, G. J., Gerards, S. & Laanbroek, H. J. (1992). Kinetics of nitrite oxidation in two Nitrobacter species grown in nitrite-limited chemostats. *Arch. Microbiol.* 157: 436-441.

Laanbroek, H. J., Bodelier, P. L. E. & Gerards, S. (1994). Oxygen consumption kinetics of Nitrosomonas europaea and Nitrobacter hamburgensis grown in mixed continuous cultures at different oxygen concentrations. *Arch.*

Microbiol. 161: 156-162.

References for *Nitrospira*:

Blackburne, R., Vadivelu, V.M., Yuan, Z.G. & Keller, J. (2007). Kinetic characterisation of an enriched Nitrospira culture with comparison to Nitrobacter. *Water Res.* 41: 3033-3042.

Gruber-Dorninger, C., Pester, M., Kitzinger, K., Savio, D.F., Loy, A., Rattei, T., Wagner, M. & Daims, H. (2015). Functionally relevant diversity of closely related *Nitrospira* in activated sludge. *ISME J.* 9:643-55.

Kim, D. J. & Kim, S. H. (2006). Effect of nitrite concentration on the distribution and competition of nitrite-oxidizing bacteria in nitratation reactor systems and their kinetic characteristics. *Water Res.* 40: 887–894.

Maixner, F. *et al.* (2006). Nitrite concentration influences the population structure of Nitrospira-like bacteria. *Env. Microbiol.* 8: 1487-1495.

Manser, R., Gujera, W. & Siegrista, H. (2005). Consequences of mass transfer effects on the kinetics of nitrifiers. *Water Res.* 39: 4633–4642.

Nowka, B., Daims, H. & Spieck, E. (2015). Comparison of oxidation kinetics of nitrite-oxidizing bacteria: nitrite availability as a key factor in niche differentiation. *Appl. Environ. Microbiol.* 81:745-53.

Schramm, A. *et al.* (1999). Microscale distribution of populations and activities of *Nitrosospira* and *Nitrospira* spp. along a macroscale gradient in a nitrifying bioreactor: quantification by in situ hybridization and the use of microsensors. *Appl. Env. Microbiol.* 65: 3690-3696.

Supplementary Table S2. Results of pairwise comparisons (1-way ANOSIM) of the genetic structures of the *Nitrobacter*-like NOB community between global change scenarios. Treatments are elevated CO_2 (CO2), increased precipitation (W), N addition (N), and all their possible combinations. CTRL corresponds to the control treatment where all factors are at ambient level. Significant differences ($p < 0.05$) and marginally significant differences (p ranging from 0.05 to 0.1, sometimes restricted by the possible permutation number due to replicate number) are indicated in bold. NS means no significant differences. The overall treatment effect is significant (R=0.24 ; p=0.001).

Treatments compared	R statistics	Significance level	Number of possible permutations
**CO2NW, CTRL**	**1**	**0.018**	**56**
**CO2NW, N**	**1**	**0.10**	**10**
CO2NW, W	0	NS	35
**CO2NW, CO2**	**1**	**0.10**	**10**
**CO2NW, NW**	**0.556**	**0.10**	**10**
**CO2NW, CO2N**	**0.856**	**0.018**	**56**
**CO2NW, CO2W**	**0.593**	**0.10**	**10**
**W, CTRL**	**0.569**	**0.016**	**126**
**W, N**	**0.481**	**0.057**	**35**
**W, CO2**	**0.435**	**0.086**	**35**
**W, CO2N**	**0.413**	**0.032**	**126**
W, CO2W	0.333	NS	35
W, NW	0.222	NS	35
N, CTRL	-0.067	NS	56
N, CO2	-0.111	NS	10
N, CO2N	-0.067	NS	56
N, NW	0.074	NS	10
N, CO2W	0.037	NS	10
CO2, CTRL	-0.108	NS	56
CO2, CO2N	-0.2	NS	56
CO2, CO2W	-0.037	NS	10
CO2, NW	0.019	NS	10
**NW, CTRL**	**0.272**	**0.089**	**56**
NW, CO2N	0.087	NS	56
NW, CO2W	-0.111	NS	10
CO2N, CTRL	0.028	NS	126
CO2N, CO2W	0.118	NS	56
CO2W, CTRL	0.21	NS	56

SUPPLEMENTARY FIGURES

Supplementary Figure S1. Overall view of the trait-based modelling approach used to complement traditional molecular approaches, and understand and predict global change effects on soil bacteria. Here, molecular approaches allowed identifying the effects of global change treatments on nitrite-oxidizing bacteria (NOB) community structure (assessed by Multi-Dimensional Scaling, see also Table S2) and on the abundances of the main NOB groups (bar graphs). A functional trait-based modelling approach was used to represent the diversity of soil NOB as three functional groups and predict NOB response to multifactorial global change. Means are presented with standard errors (n=6) for the observed responses of abundances of *Nitrobacter*-like NOB (blue bars - Top), *N. hamburgensis*-like NOB (blue hatched bars), and *Nitrospira* (blue bars - Bottom). Treatments N, W and CO2 refer to nitrogen addition, altered precipitation, and atmospheric CO_2 elevation, respectively. CTRL: control (ambient conditions).

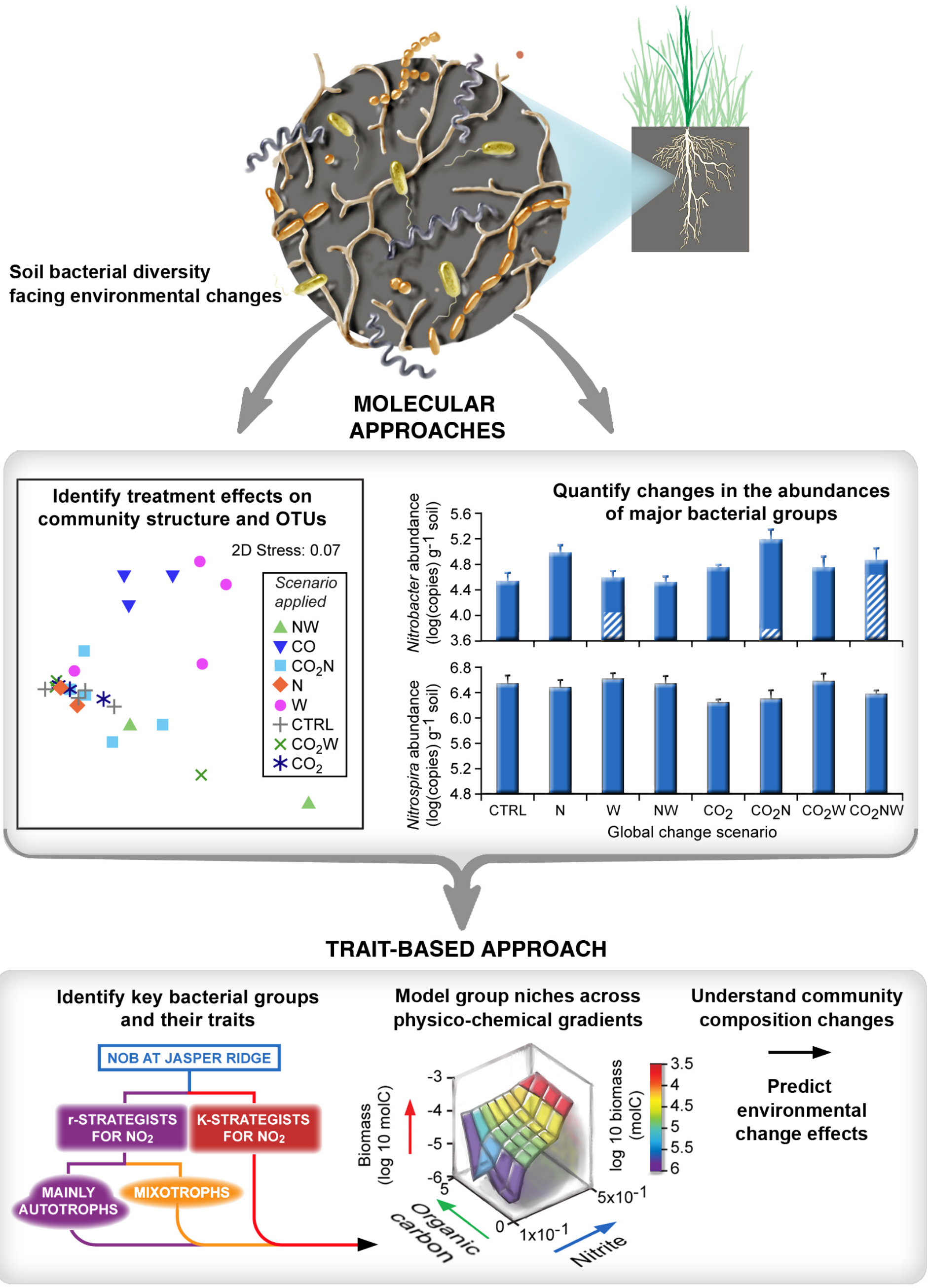
Soil bacterial diversity
facing environmental changes
MOLECULAR
APPROACHES
Identify treatment effects on
community structure and OTUs
2D Stress: 0.07
Scenario
applied
NW
CO
CO2N
N
W
CTRL
CO2W
CO2
Quantify changes in the abundances
of major bacterial groups
Nitrobacter abundance
(log(copies) g-1 soil)
Nitrospira abundance
(log(copies) g-1 soil)
CTRL
N
W
NW
CO2
CO2N
CO2W
CO2NW
Global change scenario
TRAIT-BASED APPROACH
Identify key bacterial groups
and their traits
NOB AT JASPER RIDGE
r-STRATEGISTS
FOR NO2
K-STRATEGISTS
FOR NO2
MAINLY
AUTOTROPHS
MIXOTROPHS
Model group niches across
physico-chemical gradients
Biomass
(log 10 molC)
log 10 biomass
(molC)
Organic
carbon
Nitrite
Understand community
composition changes
Predict
environmental
change effects

Supplementary Figure S2. Observed responses of potential nitrite oxidation, PNO, in response to the different global change scenarios in April 2006. Means are presented with standard errors (n = 6). N, W and CO2 refer to nitrogen addition, increased precipitation, and elevated atmospheric CO_2, respectively. CTRL refers to the control treatment where all factors are at ambient levels. Results of statistical analyses of treatment effects are presented in Table 1.

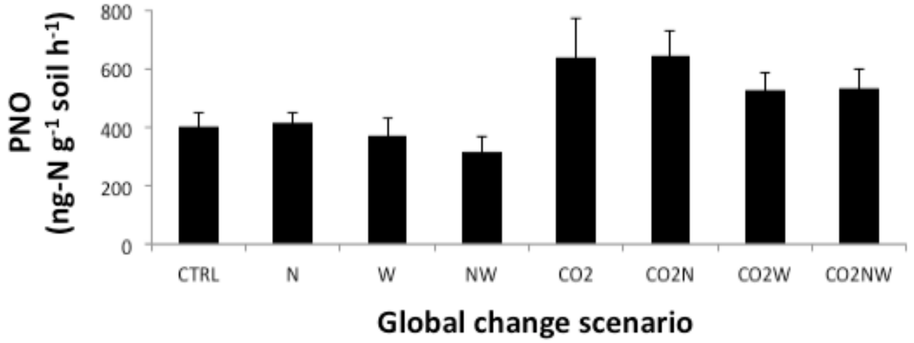

Supplementary Figure S3. Observed responses of the abundance of total *Nitrobacter*-like NOB and *Nitrospira* in response to the different global change scenarios in April 2006. Means are presented with standard errors (n=6). N, W and CO2 refer to nitrogen addition, increased precipitation, and elevated atmospheric CO_2, respectively. CTRL refers to the control treatment where all factors are at ambient levels. Results of statistical analyses of treatment effects are presented in Table 1

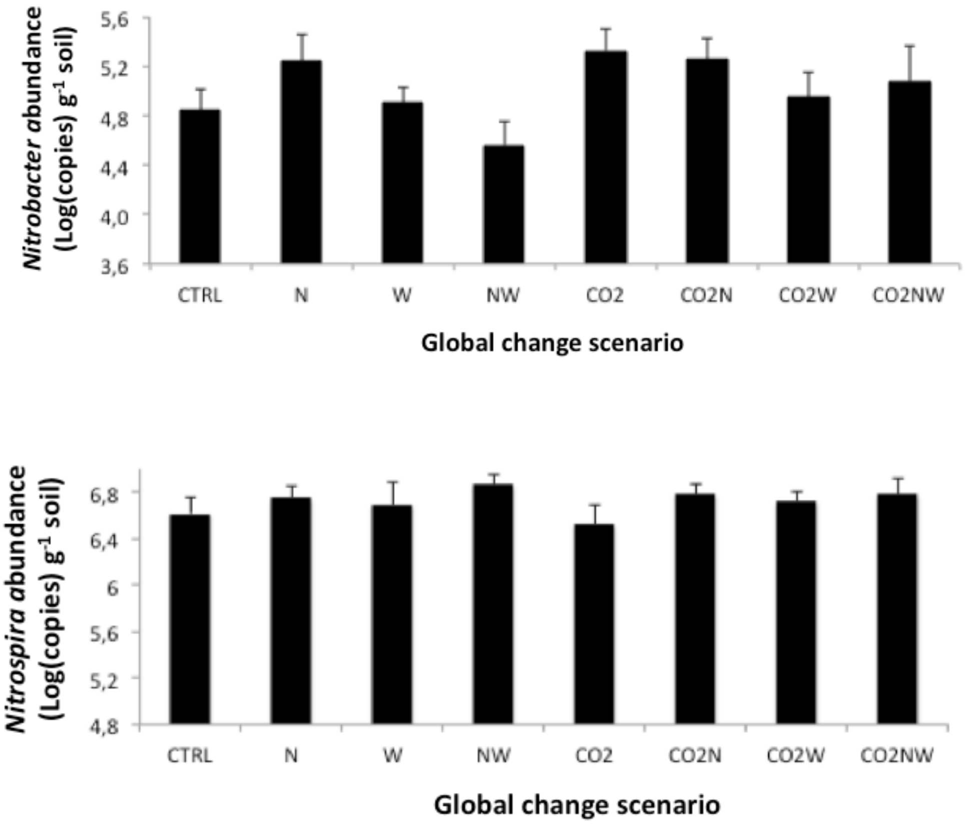

Supplementary Figure S4. Relationship between the abundance of *Nitrobacter* (•) and *Nitrospira* (o) and gross ammonification for April 2005 data. Each point corresponds to the mean value (n=6 plots) for a global change treatment. Each relationship is significant ($p<0.005$ and $p<0.001$ for *Nitrospira* and *Nitrobacter*, respectively).

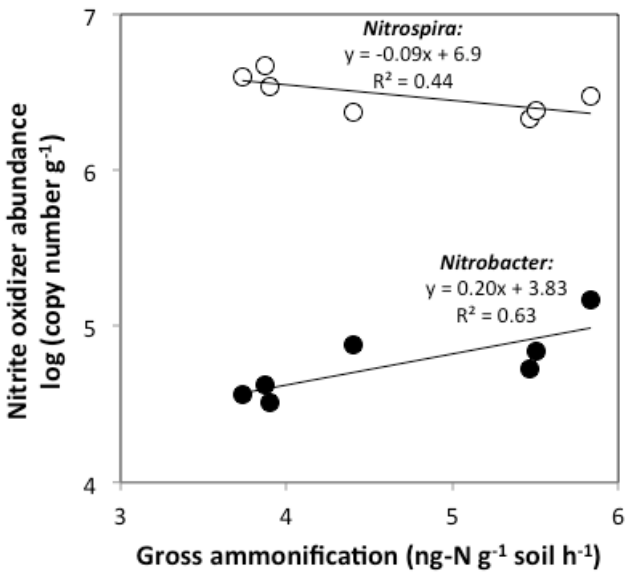

Supplementary Figure S5. Abundance of *Nitrobacter*-like NOB (Top) that do not belong to Cluster A, and (Bottom) belonging to Cluster A, i.e. related to *N. hamburgensis*, as a function of the index of soil organic carbon, OC, availability. Bars are standard errors. For *Nitrobacter*-like NOB belonging to Cluster A, the overall effect of OC availability was significant ($p<0.0005$), and different letters indicate significant differences ($p <0.05$) between treatments. Note that Y axis has a logarithmic scale.

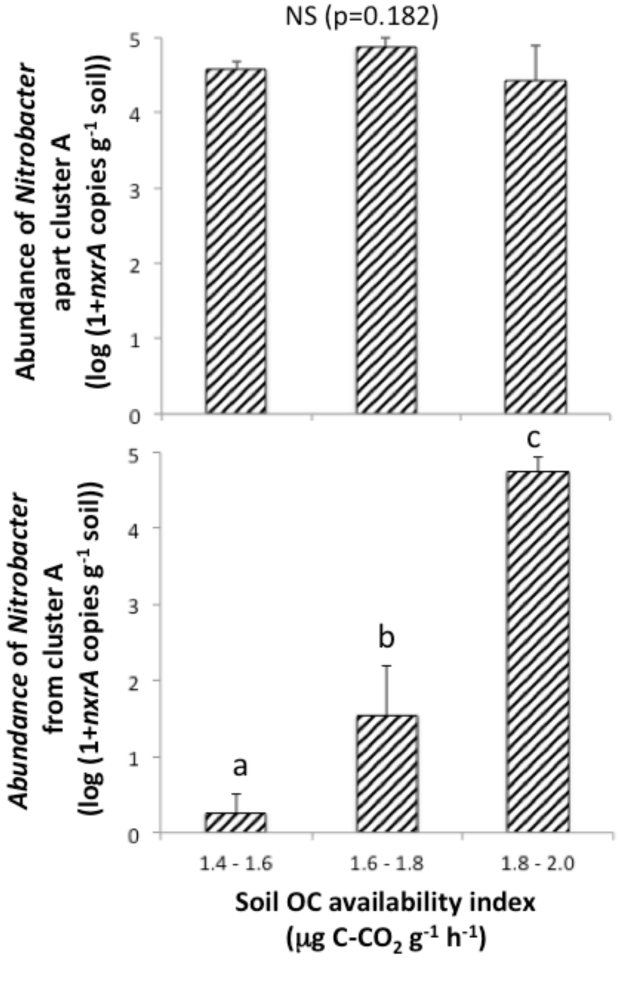

Supplementary Figure S6. Observed and predicted relative contributions of the different NOB groups for the simulations used for sensitivity analysis, i.e. distinguishing *Nitrospira* performing best as chemolitotrophs and *Nitrospira* performing best as mixotrophs in addition to the two *Nitrobacter* functional types. Observations (X-axis) are mean values with standard errors for each global change treatment. Orange circle: CTRL ; purple triangle: W ; black triangle: NW ; green circle: CO2 ; red circle: CO2N ; white circle: CO2W ; blue circle: CO2NW. The panel c thus represents the relative contribution of *Nitrospira* performing best as chemolitotrophs plus *Nitrospira* performing best as mixotrophs.

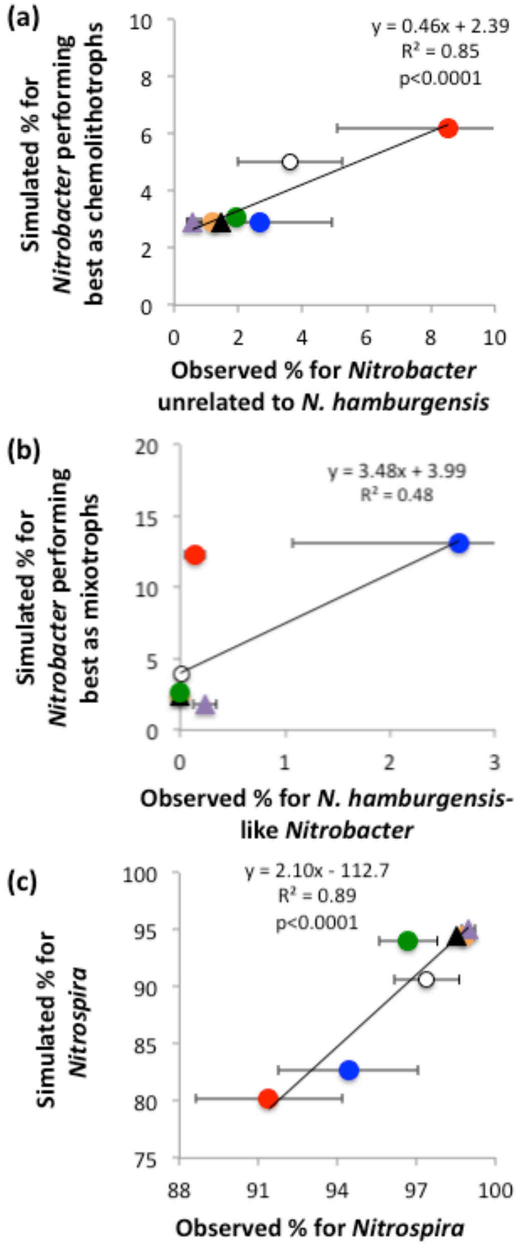
(a)
Simulated % for *Nitrobacter* performing best as chemolithotrophs
y = 0.46x + 2.39
R² = 0.85
p<0.0001
Observed % for *Nitrobacter* unrelated to *N. hamburgensis*
(b)
Simulated % for *Nitrobacter* performing best as mixotrophs
y = 3.48x + 3.99
R² = 0.48
Observed % for *N. hamburgensis*-like *Nitrobacter*
(c)
Simulated % for *Nitrospira*
y = 2.10x - 112.7
R² = 0.89
p<0.0001
Observed % for *Nitrospira*

Supplementary Figure S7. Simulated actual nitrite oxidation compared to observed potential nitrite oxidation when using the trait-based model with four functional types of nitrite-oxidizing bacteria, i.e. distinguishing *Nitrospira* performing best as chemolitotrophs and *Nitrospira* performing best as mixotrophs in addition to the two *Nitrobacter* functional types. Each point corresponds to one global change scenario, and observed mean values are presented with standard errors. Symbols are as in Figure S8.

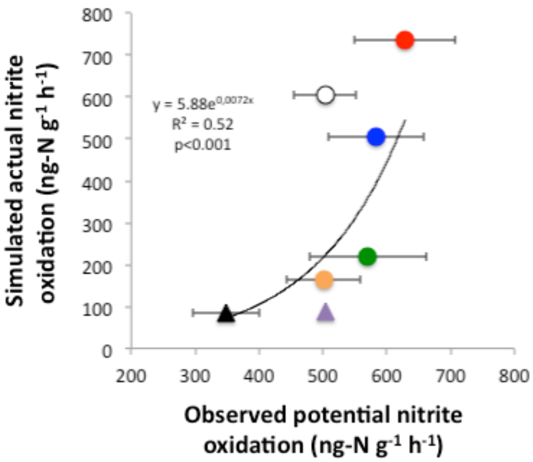

Supplement: Supplementary file 1 [file Presentation_1.PDF]
